# Characterization of *Hemerocallis citrina* Transcriptome and Development of EST-SSR Markers for Evaluation of Genetic Diversity and Population Structure of *Hemerocallis* Collection

**DOI:** 10.3389/fpls.2020.00686

**Published:** 2020-06-11

**Authors:** Sen Li, Fangfang Ji, Feifan Hou, Huliang Cui, Qingqing Shi, Guoming Xing, Yiqun Weng, Xiuping Kang

**Affiliations:** ^1^College of Horticulture, Shanxi Agricultural University, Taigu, China; ^2^Collaborative Innovation Center for Improving Quality and Increase Profits of Protected Vegetables in Shanxi, Taigu, China; ^3^Agricultural Research Service, United States Department of Agriculture (USDA-ARS), Vegetable Crops Research Unit, Horticulture Department, University of Wisconsin, Madison, WI, United States

**Keywords:** daylily, genetic diversity, *Hemerocallis*, night lily, population structure, SSR, transcriptome

## Abstract

*Hemerocallis* spp. commonly known as daylilies and night lilies, are among the most popular ornamental crops worldwide. In Eastern Asia, *H. citrina* is also widely cultivated as both a vegetable crop and for medicinal use. However, limited genetic and genomic resources are available in *Hemerocallis*. Knowledge on the genetic diversity and population structure of this species-rich genus is very limited. In this study, we reported transcriptome sequencing of *H. citrina* cv. ‘Datonghuanghua’ which is a popular, high-yielding variety in China. We mined the transcriptome data, identified and characterized the microsatellite or simple sequence repeat (SSR) sequences in the expressed genome. From ∼14.15 Gbp clean reads, we assembled 92,107 unigenes, of which 41,796 were annotated for possible functions. From 41,796 unigenes, we identified and characterized 3,430 SSRs with varying motifs. Forty-three SSRs were used to fingerprint 155 *Hemerocallis* accessions. Clustering and population structure analyses with the genotypic data among the 155 accessions reveal broader genetic variation of daylilies than the night lily accessions which form a subgroup in the phylogenetic tree. The night lily group included accessions from *H. citrina*, *H. lilioasphodelus*, and *H. minor*, the majority of which bloom in the evening/night, whereas the ∼100 daylily accessions bloomed in the early morning suggesting flowering time may be a major force in the selection of night lily. The utility of these SSRs was further exemplified in association analysis of blooming time among these accessions. Twelve SSRs were found to have significant associations with this horticulturally important trait.

## Introduction

*Hemerocallis* species are among the most popular ornamental crops worldwide because of the large, conspicuous flowers and their adaptation to a wide range of soils and climates. In Eastern Asian countries, some species, especially *H. citrina* (night lily, or long yellow daylily) are important vegetables or medicinal plants with a long history of cultivation. For example, the first reference of *H. fulva* (daylily) was found in a writing in the Chou Dynasty of China (*ca.* 112–255 BCE) (Hu, 1968; Kitchingnan, 1985). The unopened flower buds (fresh or dried) of the night lily are consumed as a special vegetable (also known as “golden needle vegetable”). The medicinal value of *Hemerocallis* species has received more attention in recent years. The flower buds seem to be enriched with antioxidants, such as stelladerol and caffeoylquinic acid derivatives (Cichewicz and Nair, 2002; Hsu et al., 2011; Lin et al., 2011; Jiao et al., 2016). These secondary metabolites are used to treat anxiety and swelling (Uezu, 1998; Cichewicz et al., 2002; Gu et al., 2012; Yi et al., 2012; Du et al., 2014; Liu et al., 2014), and for other applications in modern medicine and biology (Venugopalan and Srivastava, 2015). Thus, *Hemerocallis* flowers have considerable potentials as “nutraceutical” or “functional” foods (Rop et al., 2011).

In addition to their economic and medical values, some features of *Hemerocallis* species are of particular biological significance. For example, the day and night lilies provide an excellent model for understanding the genetic and molecular mechanisms of flower opening and flower senescing. All known species in this genus show strict circadian rhythm of flowering with the rapid opening and withering that lasts only a few hours indicting precise regulation of floral death probably by a programmed cell death system (Hasegawa et al., 2006; Nitta et al., 2010; Hirota et al., 2012, 2013). All *Hemerocallis* species exhibit self-incompatibility. As such, *Hemerocallis* was proposed as a future model system to study these biologically interesting phenomena (Rodriguez-Enriquez and Grant-Downton, 2012).

**FIGURE 1:**
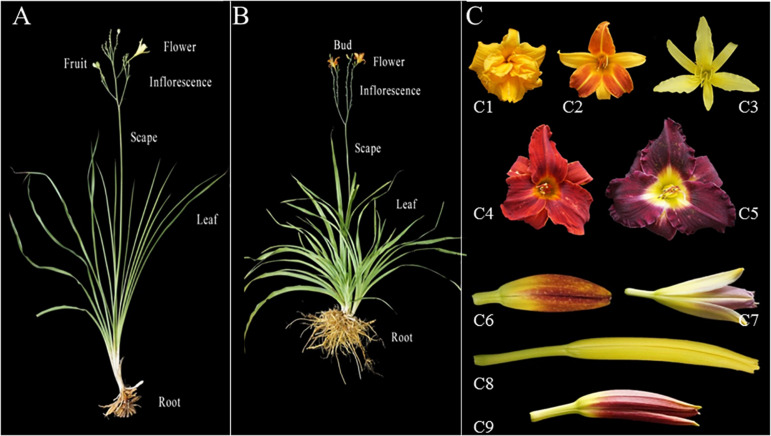
Phenotypic diversity in flowers, petals, sepals, and buds of two typical *Hemerocallis* species: *H. citrina* (night lily; **A**), and *H. fulva* (daylily, **B**). **(C)** Flower buds and opened flowers from different accessions. C1–C5 are opened flowers. C1: *Hemerocallis* cv. ‘Stella de Oro 2’; C2: *Hemerocallis* cv. ‘Frans Hals’; C3: *H. citrina* cv. ‘Datonghuanghua’ C4: *Hemerocallis* cv. ‘Blazing sun’; C5: *Hemerocallis* cv. ‘H400’; C6-C9 are young flower buds. C6: *Hemerocallis* cv. ‘Beijing 7’; C7: *Hemerocallis* cv. ‘Blue Sheen 1’; C8: *H. citrina* cv. ‘Zaohuanghua’; C9: *Hemerocallis* cv. ‘Little Grapette’.

As an ornamental plant, extensive breeding efforts during the last century have resulted in varieties with different flower size, color, shape, scent and blooming time. In the US, over 83,000 daylily varieties have been registered in the American *Hemerocallis* Society online database^1^. Some morphological characteristics of daylily and night lily plants and flowers are exemplified in Figure 1. Despite the economic and biological importance as well as extensive breeding efforts on *Hemerocallis* species, not much has been accomplished on genetic studies in this genus. As demonstrated in numerous other horticulture crops, molecular markers are important tools for genetic research and breeding in *Hemerocallis* with many potential applications such as evaluation of genetic diversity and population structure of germplasm collection, development of linkage maps, gene and QTL (quantitative trait loci) mapping or cloning, as well as marker-assisted selection of horticulturally important traits in breeding. Nevertheless, limited work has been done in the development of molecular markers for *Hemerocallis* species. The natural range of *Hemerocallis* encompasses temperate and sub-tropical Asia, with the main center of diversity of the genus in China, Korea and Japan (Flora of China, 2000). However, reports on the genetic diversity and population genetic analysis of *Hemerocallis* especially for the collections in China are sporadic (e.g., Tomkins et al., 2001; Podwyszynska et al., 2009; Vendrame et al., 2009; Miyake and Yahara, 2010). In these early reports, molecular markers employed included random amplified polymorphic DNA (RAPD) and amplified fragment length polymorphism (AFLP), each of which has limitations in practical uses (Varshney et al., 2007). Despite the increased use of single nucleotide polymorphism (SNPs), SSR markers remain popular in plant genetic and breeding studies because of their many desirable attributes, including hyper variability, a multi allelic nature, co-dominant inheritance, reproducibility, relative abundance, extensive genome coverage (including organellar genomes), relatively low cost, and amenability to high throughput genotyping (Parida et al., 2009). However, very few SSRs have been reported for *Hemerocallis* (e.g., Zhu et al., 2009). While there are several methods to develop SSR markers, transcriptome sequencing (RNA-Seq) has been an efficient and affordable way for large-scale, and genome-wide SSR discovery and marker development for various marker-based studies in non-model plants (e.g., Hudson, 2010; Strickler et al., 2012; Yuan et al., 2013; Zhang et al., 2013; Guo et al., 2016; Smithunna et al., 2016). Thus, the objective of the present study is to develop EST (expressed sequence tag)-SSR markers through transcriptome sequencing and examine their utility in evaluation of genetic diversity and population structure of our *Hemerocallis* collection. We also conducted association analysis to identify potential association of molecular markers with horticulturally important traits.

## Materials and Methods

### Plant Materials

**FIGURE 2:**
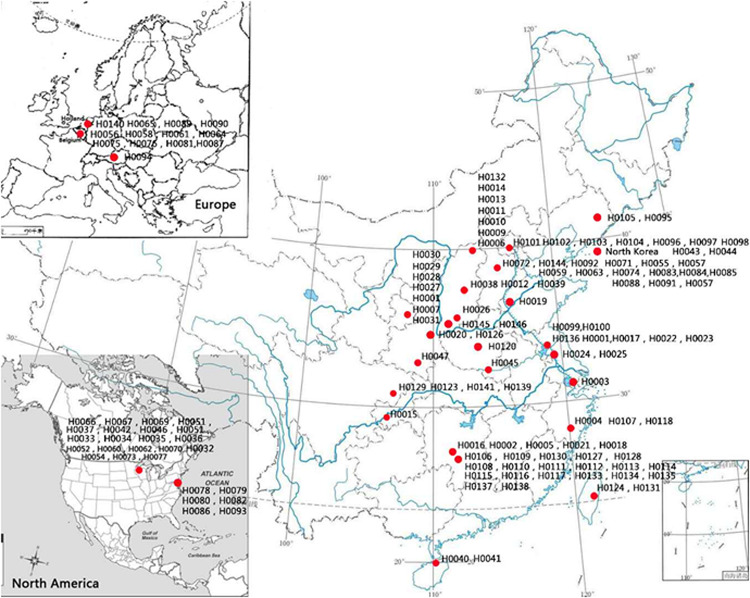
Geographic distribution 155 *Hemerocallis* accessions used in this study.

One hundred and fifty-five *Hemerocallis* accessions were employed for genetic diversity analysis with 43 EST-SSRs. These accessions belong to at least 13 species from different geographic regions around the world including commercial varieties, landraces, and collections from the wild. The details of these 155 accessions are presented in Supplementary Table S1, and their geographic distributions are illustrated in Figure 2. The taxonomic status of more than half of the 155 accessions was labeled uncertain during collection (*Hemerocallis* spp. in Supplementary Table S1). All accessions were grown in the *Hemerocallis* Germplasm Resource Nursery located on the campus of Shanxi Agricultural University (Taigu, Shanxi Province, China). All plants used for sample collection have been grown for 3–5 years in the nursery. At full flowering stage, the fresh roots, flower buds, and leaves of the *H. citrina* cv ‘Datonghuanghua’ were collected, flash frozen in liquid nitrogen, and used for total RNA extraction and RNA-Seq.

### RNA-Seq, Transcriptome Assembly, and Annotation

For RNA-Seq, total RNA was isolated from each sample with three plants pooled with a modified CTAB protocol (Tel-Zur et al., 1999). RNA quantity and quality were evaluated using the Agilent Bioanalyzer (Agilent Technologies, CA, United States). The integrity of the total RNA was also assessed through agarose gel electrophoresis. The cDNA library for RNA-Seq was prepared using Illumina Truseq^TM^ RNA sample prep kit (Illumina, CA, United States) following manufacturer’s protocols. The concentration and insert size of the library were assessed using Qubit2.0 and Agilent 2100. Pair-end (125 PE) Illumina high-throughput sequencing was performed with a Hi-Seq 2500 sequencing machine. All samples were sequenced in the same instrument (HWI-7001455), same run (316), same follow cell (HH7VHADXX), and same lane (2).

After resequencing, the adaptors and low quality reads were filtered from the raw reads. The clean reads were *de novo* assembled into contigs with an optimized k-mer length = 25 and group pairs distance = 300 (Grabherr et al., 2011) using the Trinity program^2^. Unigene sequences were aligned against databases of the Clusters of Orthologous Groups (COG) (Tatusov et al., 2000), Gene Ontology (GO) (Michael et al., 2000), Kyoto Encyclopedia of Genes and Genomes (KEGG) (Kanehisa et al., 2004), euKaryotic Orthologous Groups (KOG) (Eugene et al., 2004). The functions of unigenes were predicted by BLAST against the NCBI non-redundant protein (Nr), and Swiss-Prot databases at an *E*-value of 10^–5^. The resulting datasets were further aligned to the Protein family (PFAM) database with HMMER (*E*-value 10^–10^). For further quantitative assessment of the completeness of the assembly and annotation, the *H. citrina* cv ‘Datonghuanghua’ transcriptome was subjected to BUSCO (Benchmarking Universal Single-Copy Orthologs) analysis (Simão et al., 2015) against the Viridiplantae_odb10, embryophyta_odb10, and liliopsida_odb10 databases.

### Microsatellite Sequence Identification and EST-SSR Genotyping

SSR identification among the unigene sequences (>1 kb) was performed with the MIcroSAtellite (MISA) program^3^. All microsatellites containing di-, tri-, tetra-, penta-, hexa-nucleotide, and compound motifs with more than five repeats were included. The Primer3 program^4^ was used to design primers for the identified SSRs. Each designed primer pair was first evaluated with *in silico* PCR procedure^5^ using unigene sequences as the template. Primers with multiple amplicons were filtered out.

Forty-three EST-SSR markers were selected for genotyping 155 *Hemerocallis* accessions. Genomic DNA was extracted from fresh leaves of each accession (pooled from five plants) using the CTAB method. A touch-down PCR procedure (Azhaguvel et al., 2009) was employed and the amplicons were separated using 9% non-denaturing polyacrylamide gel electrophoresis (PAGE) with 0.5 × TBE buffer for 1.5 h in 250V, and then visualized with silver staining.

### Cluster and Population Structure Analyses

The SSR genotypic data were organized in a matrix in which 0 and 1 representing absent and present alleles, respectively (9 = missing data). The genetic distances were calculated through the SM coefficient by the *Sim Qual* procedure in NTSYSpc 2.10 (Rohlf and Jensen, 1989). The dendrogram was constructed using the unweighted pair-group method with arithmetic mean (UPGMA) clustering and drawn by NTSYSpc 2.10. We also applied Neighbor Joining (NJ) clustering on the dataset, which was implemented in the MEGA5.05 software package.

A model-based Bayesian clustering was applied to infer genetic structure and define the number of clusters (gene pools) in the dataset using STUCTURE v.2.3.4 (Pritchard et al., 2000). No prior information was used to define the clusters. Independent runs were done by setting the number of clusters (*K*) from 1 to 15. Each run was comprised of a burn-in length of 10,000 followed by 100,000 MCMC (Monte Carlo Markov Chain); each replicate at a particular *K*-value was repeated 20 times. An *ad hoc* statistic Δ*K* based on the rate of changes in the log probability of data among successive *K-*values (Evanno et al., 2005) was calculated through Structure Harvester v.0.9.93 and used to estimate the most likely number of clusters (*K*). Δ*K* was calculated using Δ*K* = m[| L(*K*+1)-2L(*K*)+L(*K*-1)|]/s| L(*K*)|, where (L(*K*) is the logarithm of *K*, s is the standard deviation, and m is the mean.

### Association Analysis for Blooming Time in 155 *Hemerocallis* Accessions

We performed association analysis of blooming time with SSR markers among the 155 accessions of the *Hemerocallis* collection. During flowering season, the time of flower opening was continuously monitored real-time using a digital video camera (360 Smart Camera, D606, 360 China). The flowering time of each plant from each accession was recorded. The blooming time of at least five flowers per accession was collected. The blooming time of each accession is provided in Supplementary Table S1. In association analysis, the blooming time was treated a qualitative trait. Accessions with blooming time from 4:00 to 10:00 and 16:00 to 24:00 were assigned “0” and “1,” respectively.

Pairwise linkage disequilibrium (LD) and association analyses were performed using TASSEL4.3.6 (Bradbury et al., 2007; Dent et al., 2012), and calculation of LD and *P*-values. Association analysis was performed using both the general linear model (GLM, Q model) and the mixed linear model (MLM, Q+K model) in TASSEL. The comparison wise significance was computed using 1,000 permutations as implemented in GLM. The kinship matrix was generated by NTSYSpc 2.10 with option P3D for variance component estimation in MLM (Kang et al., 2008). Significance of marker-trait associations was determined at *P* ≤ 0.05 (Jaiswal et al., 2012). False discovery rate (FDR) was also used to detect true associations.

### Gene Expression in Different Tissues of *H. citrina* cv. ‘Datonghuanghua’

The expression level of blooming time-related gene *c33464.graph_c0* in different tissues (fresh roots, flower buds and leaves) of *H. citrina* cv. ‘Datonghuanghua’ (H0006) was carried out with quantitative real-time PCR (RT-qPCR) on an ABI prism 7500 Fast Real-time PCR system 147 machine (Thermo Fisher Scientific Inc., Waltham, MA, United States). Sequences of the primers for *c33464.graph_c0* were GGCGAATTAGTCTGGAAAGAACTAGG (forward primer) and TGTTATGTTCCTCGTCCGTCCAC (reverse primer). The *H. citrina actin* gene, *HcACT*, was used as the reference. The primers sequences for this gene were GAGCAAGGAAATCACGGCACT (forward primer) and GGAACCTCCAATCCAAACACTGTAC (reverse primer). RT-PCR procedure followed Hou et al. (2017). Each sample had three biological and three technical replications.

## Results

### Transcriptome Sequencing, *de novo* Assembly, and Unigenes Annotation

**FIGURE 3:**
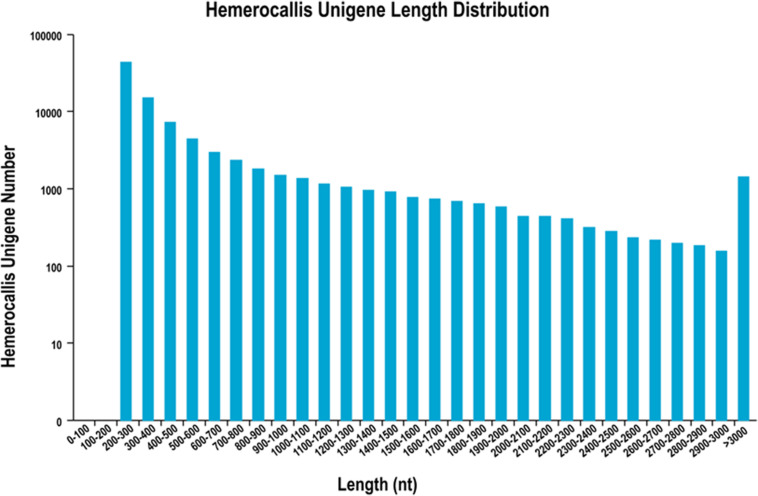
Length distribution of unigenes from leaf, root and flower bud transcriptomes of *H. citrina* cv. ‘Datonghuahua’.

After filtering of the raw sequencing data, nearly 56 million clean reads (14.15 Gb) were obtained from root, bud and leaf transcriptomes. Main RNA-Seq statistics for the three tissues are presented in Supplementary Table S2. In all three transcriptomes, >90% reads had Q30 or higher quality scores. Using the Trinity *de novo* assembly program, the ∼14.15 Gb high-quality reads were assembled into contigs, transcripts and unigenes. Main statistics of the assemblies are shown in Table 1. From 6,390,477 contigs (>25 bp in length), 164,723 transcripts were assembled (mean length 838 bp) representing 92,107 unigenes. The length distribution of these unigenes is illustrated in Figure 3. The transcripts with >500 bp in length accounted for 47.06% of all transcripts. There were 13,724,749 (76.9%), 15,731,521 (79.2%), and 14,841,142 (80.4%) reads from the roots, buds and leaves mapped to the assembly transcripts and unigenes, respectively.

**TABLE 1:** Transcriptome assembly statistics of *H. citrina* cv. ‘Datonghuanghua’.

Length range (bp)	Contigs (%)	Transcripts (%)	Unigenes
200–300	6,332,326 (99.09%)*	53,717 (32.61%)	43,822 (47.58%)
300–500	28,381 (0.44%)	33,471 (20.32%)	22,209 (24.11%)
500–1000	15,997 (0.25%)	31,124 (18.89%)	12,983 (14.10%)
1000–2000	9,538 (0.15%)	30,756 (18.67%)	8,806 (9.56%)
>2000	4,235 (0.07%)	15,655 (9.5%)	4,287 (4.65%)
Total number	6,390,477	164,723	92,107
Total length	328,357,742	138,156,556	52,976,189
N50 length	48	1,467	908
Mean length	51.38	838.72	575.16

*Indicates the numbers and percentage of contigs with length between 200 and 300 bp.

We annotated the 92,107 unigenes by searches against the COG, GO, KEGG, KOG, Nr, and Swiss-Prot databases. Of them, 41,796 (45.4%) had hits in all 7 databases. The unigene hits in each database are shown in Table 2 with the number of annotated unigenes being the highest in the NR (41,053) and the lowest in KEGG (9,375). Further BLAST searches against other databases showed that 12,537, 23,716, 23,393, 28,711, and 24,125 unigenes had at least one match in the COG, GO, KOG, Swiss-Prot and PFAM databases, respectively (Table 2). In BLASTx homology searches (at cutoff *E*-value of 10^–5^), the ten top species with hits in the Nr database were: *Elaeis guineensis* (8,779; 21.38%), *Phoenix dactylifera* (7,194; 17.52%), *Musa acuminate* (2,912; 7.09%), *Nelumbo nucifera* (2,273; 5.54%), *Vitis vinifera* (2,204; 5.37%), *Populus euphratica* (761; 1.85%), *Oryza sativa* (650; 1.58%), *Citrus sinensis* (582; 1.42%), *Populus trichocarpa* (554; 1.35%), and *Hordeum vulgare* (474; 1.15%).

**TABLE 2:** Number of unigenes with annotations in six databases of *H. citrina* cv. ‘Datonghuanghua’.

	COG	GO	KEGG	KOG	Nr	Swiss-Prot	Pfam	Total
# Unigenes	12,537	23,716	9,375	23,393	41,053	28,711	24,125	41,796
≥300 nt	9,517	16,309	6,580	16,434	28,548	21,038	19,080	28,834
≥1000 nt	5,318	7,437	3,098	7,694	12,090	9,629	10,346	12,111

**FIGURE 4:**
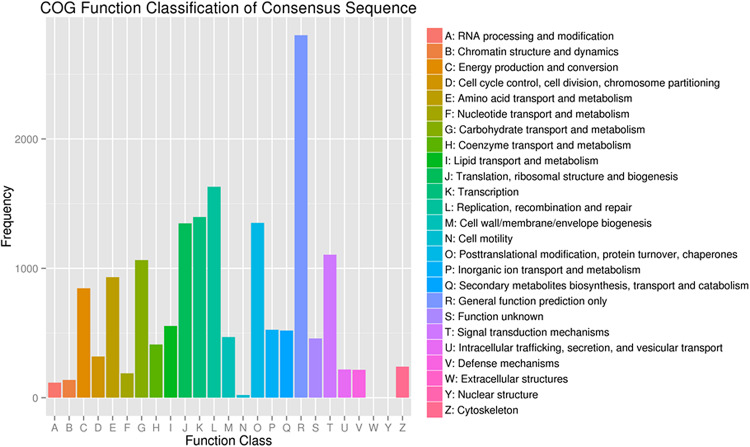
Functional classification of the assembled unigenes based on Clusters of Orthologous Groups (COG) categories.

To further evaluate the functions of unigenes, 12,537 of the assembled 41,796 unigenes were classified into 25 clusters based on the Cluster of Orthologous Groups (COG) analysis (Figure 4). Among the 25 COG categories, the cluster related to general function prediction (2,801 or 22.3%) was the largest, followed by the clusters of replication, recombination and repair (1,630 or 13.0%), transcription (1,397 or 11.1%), and secondary metabolites biosynthesis, transport and catabolism (519 or 4.1%). The clusters represented by the least number of unigenes were cell motility, nuclear structure, and extracellular structures (1, 0.008%).

**FIGURE 5:**
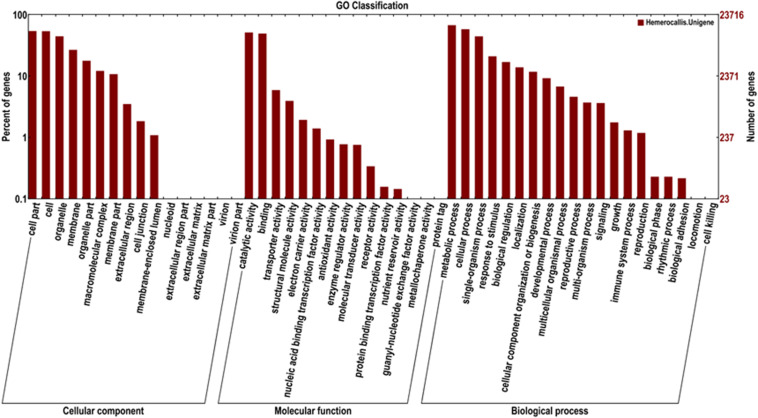
Functional classification of the assembled unigenes based on Gene Ontology (GO).

In addition, 23,716 unigenes matched with the GO database were categorized into 51 functional sub-groups of the three main GO groups: cellular component, molecular function, and biological process (Figure 5). The majority of the unigenes was assigned to biological processes (63,089, 43.7%), followed by cellular components (35,677, 37.2%), and molecular functions (27,719, 19.2%). Under the category of biological processes, metabolic process (15,976, 25.3%) and cellular process (13,749, 21.8%) were predominant. In the cellular component category, cell part (12,837, 23.9%) and cell (12,746, 23.8%) were the most abundant classes. As for the molecular function, catalytic activities (12,157, 43.9%) and binding (11,677, 42.1%) were the top two categories in numbers.

**FIGURE 6:**
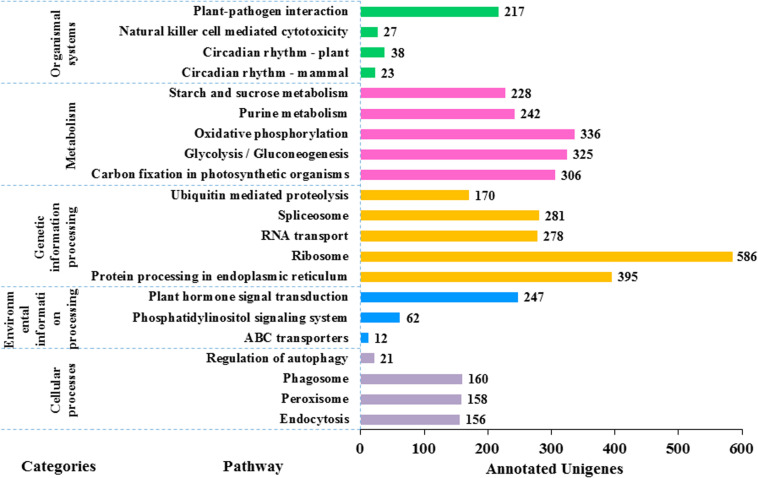
Functional classification of the assembled unigenes based on Kyoto Encyclopedia of Genes and Genomes (KEGG) pathway analysis. 9,375 unigenes are annotated to five categories: metabolism, genetic information processing, cellular processes, environmental information processing and organic systems. There are a large number of pathways of metabolism and genetic information processing (in total 106 pathways) but the picture is limited, so the first five pathways with the largest number of each category are selected for plotting. The value to the right of each bar indicates the number of unigenes annotated in the pathway.

In KEGG pathway analysis of the 41,796 unigenes, 9,375 (22.4%) could be assigned to 117 pathways that belong to five main categories including metabolism (6,783), genetic information processing (3,013), cellular processes (495), environmental information processing (321), and organismal systems (305) (see details in Figure 6). The colchicine content is a very important trait for commercial production of night lily. Among annotated unigenes, 20 were involved in the isoquinoline alkaloids metabolic pathway that is related with colchicine biosynthesis. The category with the largest number of unigenes was metabolism, in which the most represented five pathways were oxidative phosphorylation (ko00190) (336, 4.9%), glycolysis/Gluconeogenesis (ko00010) (325, 4.8%), carbon fixation in photosynthetic organisms (ko00710) (306, 4.5%), purine metabolism (ko00230) (242, 3.6%), and starch and sucrose metabolism (ko00500) (228, 3.4%).

We conducted BUSCO analysis to evaluate completeness of the transcriptome using the near-universal orthologous gene groups. The details are presented in Supplementary Table S3 and illustrated in Supplementary Figure S1. For example, among the 430 BUSCOs in the viridiplantae_odb10 database, *H. citrina* cv. ‘Datonghuanghua’ transcriptome had 388 (90.2%) complete single or duplicated copies, 27 were fragmented, and 15 were missing, which indicated that this transcriptome assembly had adequate coverage and quality to represent the *H. citrina* transcriptome for various downstream analyses.

### Identification and Characterization of EST-SSRs in Datonghuahua Transcriptomes

Using MISA, we mined microsatellite-containing sequences from 13,019 unigenes with >1,000 bp in length. As a result, 3,430 potential SSRs were identified from 2,977 unigenes, 453 of which contained more than one SSR (detailed in Supplementary Table S4). Details of the 2,977 unigenes, associated SSRs, and motif statistics are provided in Supplementary Tables S5, S6, respectively. The sequences of these unigenes in fasta format are provided in Supplementary Table S7. Among those SSRs, those with the tri-nucleotide repeat motifs were the most abundant (54.29%, 1,862), followed by SSRs with di-nucleotide (31.75%, 1,089), and tetra-nucleotide (3.24%, 111) motifs. Other types were rare. Among the di- and tri-nucleotide repeats, (GAA/TTC)_*n*_, and GA/TC)_*n*_ or (AG/CT)_*n*_ were the most common repeat motifs, respectively (Supplementary Table S6).

From the 2,977 SSR-containing unigene sequences, primers were designed for 3,577 SSRs. The primer sequences for each SSR and associated information are provided in Supplementary Table S8. From the list, we tested 192 experimentally in six accessions. Based on the preliminary data, we finally selected 43 SSRs that had clear, and unique amplicons with expected size. Information for the 43 EST-SSRs is provided in Table 3.

**TABLE 3:** Information of 43 SSRs used for assessment of genetic diversity among 155 *Hemerocallis* accessions.

No.	SSR name	SSR Motif	Forward primer sequence (5′→3′)	Tm/°C	Reverse primer sequence (5′→3′)	Tm/°C	Amplicon size (bp)
1	SAU00003	(GTC)6…(AG)8…/(AG)6	F:GTGTTGTCGGAGAAGGAGGA	60.2	R:CCAATTCGTTCACATCTCCC	60.3	226
2	SAU00006	(TAG)5…(GAG)5…(AGG)6	F:CCAAGTAAGAGGGTGCATCA	58.7	R:ATGTCATCTCCATCAAGGGC	59.9	241
3	SAU00008	(GTCC)5 (CTCC)5	F:TCTTAATCGGACCATCGAGC	60.2	R:TTGAAATCGAGAGATTGGGG	60.0	137
4	SAU00009	(TCA)5…(GA)10	F:GTGGTTGATTGGAGGCTGAT	59.9	R:AGGGGTTGAGCACGTATTTG	60.0	261
5	SAU00010	(CA)9…(AC)7	F:ATACAAGCCATTGGCAAACC	59.8	R:GAAGATTCCCCCGTTGATCT	60.3	220
6	SAU00014	(TC)7…(CT)10	F:CGGAACAACATCGCCTAAAT	60.0	R:ATAAGGTTGGGGGTGAGGAG	60.2	254
7	SAU00017	(A)12…(TCT)5	F:TCTCCTATATAAAAACAAAAACGAAAA	58.1	R:TCTCACTTTGAGCTTGCGAA	59.9	248
8	SAU00029	(AT)7	F:TCACTGCAACCAAATGAAGG	59.7	R:GGGGAGGTTATGGACGAGTT	60.2	243
9	SAU00032	(CT)6	F:AATGTCCCGACTTCAGATGG	59.9	R:GCGAGGAAGGGAAGAAAAAG	60.3	260
10	SAU00042	(AAC)6	F:GGGATGAAAGAAACGCAATC	59.5	R:TGCACAAAACAATTGCATCA	59.7	183
11	SAU00045	(AAG)5	F:GGTGGAGGTGAGGTTCTCAA	60.1	R:CCAGCTTTCAAATGGACGAT	60.1	175
12	SAU00047	(GCC)5	F:AGAAGGTTGTGGATTGCTGG	60.1	R:CCTTCTCTCTCGTCTCCCCT	59.9	183
13	SAU00048	(AGT)5	F:CTCTTCGGCAGGAGTAGTGG	60.0	R:CCAGTCCATCAAAACGTCCT	60.0	151
14	SAU00052	(CTC)5	F:TGGTCTAATCATTGTCCCCC	59.6	R:TCAATCATTCAGTTGGGCTG	59.6	107
15	SAU00055	(CTC)6	F:AAATCCCCCAACCATCTTTC	60.0	R:GAGTGACGGTGGGTTTGAGT	60.0	207
16	SAU00059	(CCA)5	F:CCTCTTCTCTTCCCCTCACC	60.2	R:ATGAGCTTCACCAGGGTTTG	60.1	189
17	SAU00063	(AGA)6	F:TTGTATACGGTTTCCTCGCC	60.0	R:GTCAAATTGAGCGGTTGGAT	59.9	228
18	SAU00064	(ATCG)5	F:GCTTCTTCTTGTCAGCACCC	60.0	R:ACCGAAACCCTCGGTAAGTT	59.9	192
19	SAU00069	(GAAT)5	F:GGCAGAGGAAGAATTGTCCA	60.2	R:ATGTTCTTTCCTTCGCCTCA	59.8	218
20	SAU00080	(AGAT)5	F:TTCTCCGCCTTGGTGTACTT	59.7	R:CTCCTCAGCTTGCTTCGTCT	59.9	181
21	SAU00095	(CCGCCA)5	F:TCTCTCTCATCGCTTCCCAT	59.9	R:GGCACGGTGAGCAACTCTAT	60.3	201
22	SAU00096	(AAAGCC)5	F:ACCACATGTCCTCCTATCGC	60.0	R:TCCCGAATAGCGTGTCCATA	61.4	206
23	SAU00097	(T)10 (TG)8*	F:ATCGCATATAGCGGACCTTG	60.1	R:CCGAGACACAAAACTCACGA	59.9	112
24	SAU00102	(TGAAAC)5	F:CTCCAGTGACAGTCAGCCAA	60.0	R:TCTGTGATGCTGCTTGGTTC	60.0	130
25	SAU00104	(CTGTG)5	F:TGAATCAAACTCCACCACCA	59.9	R:GTTTGGAGATTGGAGGGGTT	60.2	221
26	SAU00109	(ATCG)5	F:GCTTCTTCTTGTCAGCACCC	60.0	R:ACCGAAACCCTCGGTAAGTT	59.9	192
27	SAU00120	(TATT)5	F:CCTTCCTCAATCCAACCTCA	60.0	R:GTCGGGGTACAATTCGAAGA	59.9	150
28	SAU00121	(GTCA)5	F:TTTGCATGCCTAGTCCTGTG	59.9	R:AAGTCGCAGACCAATTCGAT	59.7	206
29	SAU00124	(GAAG)5	F:CAACAACAAATCCCCAATCC	60.0	R:CCATGGATCAAATACCACCC	59.9	204
30	SAU00132	(GAG)6	F:ACGGAGGGTCGAGTTGTATG	60.0	R:AGTTGACGAATCTCCCGATG	60.1	184
31	SAU00135	(ACC)5	F:GGAAATTTTCGTCCGAGTCA	60.1	R:TTTGGTCACACGAAAGGACA	60.1	206
32	SAU00137	(GAA)6	F:TGAATCCGAGAGACCTTTGG	60.2	R:ACCCTCGTCCTCAAACCCT	60.9	145
33	SAU00138	(TTG)5	F:AAGCAGCATTTGAGGTTTGG	60.2	R:CCCTGAAGCTCCTCATCTTG	59.9	176
34	SAU00141	(TGG)5	F:TCGAGTTTAGGTGGAGGGTG	60.1	R:ACAGACAATGGCAACAACCA	60.0	170
35	SAU00143	(CCA)5	F:CAGAGCTGTCCTTCCTCCAC	60.0	R:AAATCGATGGGCTACAGGTG	60.0	214
36	SAU00144	(CAT)5	F:TAAACACCTCGACAGCCTCC	60.3	R:ACGAGGAAAGGAGGAGTGGT	60.1	200
37	SAU00150	(CGC)6	F:TGCTTGTCATCCTCCTTTCC	60.2	R:TATAGATCGAAGCTCGGCGT	60.0	278
38	SAU00160	(AT)6	F:AATAGGCATCCACCAGGAGA	59.5	R:CATGAACTCGGCACTCTGAA	60.0	177
39	SAU00164	(GA)6	F:GATGAGGTCGAGAGGATGGA	60.2	R:AAAACCGATACCCAGAACCC	60.1	262
40	SAU00171	(TC)11…(CT)8	F:TAGTCATTACCTTCGCCGCT	59.9	R:AATGAGCGGTTTTCCATGAC	59.9	230
41	SAU00172	(GTG)6…(GTG)5	F:CATTTTTGGTGTGCCTGTTG	60.0	R:TCCACAAAATACAAATTGCAAAA	59.4	185
42	SAU00176	(GGT)5…(GGA)6	F:AGGATCTGGTGAGGGGAGAG	60.6	R:TAAACCAACCCCTAGGCCC	61.0	232
43	SAU00180	(CAT)5…(AGG)6	F:CTTTTGCTCCCAAATTGAGC	59.8	R:TAATGATCTTTTCGGGCACC	59.9	279

### Genetic Diversity of *Hemeracallis* Germplasm Collection

The forty-three EST-SSRs were used to fingerprint 155 *Hemeracallis* accessions. A total of 396 alleles were detected at the 43 marker loci. The number of alleles detected at each locus varied from 2 to 25 with an average of 9.21 per locus. The polymorphism information content (PIC) ranged from 0.907 to 0.259 with an average of 0.606 (Table 4). Mean Shannon’s information index (*I*) was 1.294 (0.477–2.653). Mean observed heterozygosity (*Ho*) and mean expected heterozygosity (*He*) was 0.1757 (0.0000–0.5175) and 0.5935 (0.2104–0.9109). These indicated that the 43 SSRs provided rich genetic information (Table 4).

**TABLE 4:** Estimation of genetic diversity based on 43 SSRs among 155 *Hemerocallis* accessions.

No.	SSR name	Allele number	PIC	*I*	Ho	He	Amplicon range (bp)
1	SAU00003	5	0.339	0.623	0.0654	0.2902	226–260
2	SAU00006	6	0.753	1.465	0.2680	0.7556	210–241
3	SAU00008	16	0.826	2.135	0.0263	0.8305	130–160
4	SAU00009	6	0.603	1.122	0.1290	0.6156	250–261
5	SAU00010	10	0.692	1.449	0.0968	0.6792	220–245
6	SAU00014	10	0.752	1.669	0.2973	0.7247	175–190
7	SAU00017	2	0.367	0.516	0.1181	0.3351	290–300
8	SAU00029	14	0.784	1.891	0.2230	0.7986	250–270
9	SAU00032	8	0.713	1.466	0.2000	0.7094	240–255
10	SAU00042	25	0.756	2.101	0.2914	0.7855	180–200
11	SAU00045	10	0.635	1.291	0.1457	0.6242	150–170
12	SAU00047	13	0.665	1.617	0.2649	0.7014	183–205
13	SAU00048	10	0.763	1.717	0.0592	0.7491	150–175
14	SAU00052	22	0.907	2.653	0.0704	0.9109	107–130
15	SAU00055	8	0.500	1.062	0.1067	0.4802	185–207
16	SAU00059	8	0.357	0.771	0.0567	0.3358	200–215
17	SAU00063	5	0.547	0.945	0.1382	0.5069	228–240
18	SAU00064	6	0.598	1.047	0.2752	0.5481	200–215
19	SAU00069	10	0.662	1.467	0.1026	0.6443	218–240
20	SAU00080	5	0.261	0.596	0.0000	0.2615	160–175
21	SAU00095	6	0.614	1.127	0.3333	0.5940	200–210
22	SAU00096	16	0.784	2.011	0.5175	0.8091	210–235
23	SAU00097	12	0.820	1.931	0.2465	0.8026	112–130
24	SAU00102	8	0.748	1.557	0.0403	0.7466	125–140
25	SAU00104	7	0.631	1.248	0.2017	0.6407	221–230
26	SAU00109	11	0.418	0.970	0.0336	0.4067	170–185
27	SAU00120	4	0.548	0.915	0.3517	0.5558	140–160
28	SAU00121	9	0.528	1.018	0.1522	0.4796	190–206
29	SAU00124	6	0.595	1.015	0.2039	0.5761	200–210
30	SAU00132	5	0.519	0.803	0.2763	0.5031	180–190
31	SAU00135	4	0.259	0.477	0.0851	0.2104	195–206
32	SAU00137	5	0.450	0.716	0.3311	0.3937	145–160
33	SAU00138	5	0.540	0.897	0.3007	0.4848	170–176
34	SAU00141	10	0.642	1.309	0.3226	0.6217	170–190
35	SAU00143	8	0.376	0.731	0.1234	0.3285	214–230
36	SAU00144	7	0.518	0.932	0.0420	0.5164	200–220
37	SAU00150	16	0.832	2.173	0.0355	0.8264	265–285
38	SAU00160	6	0.548	1.036	0.1141	0.5271	170–185
39	SAU00164	6	0.503	1.014	0.0000	0.5045	262–275
40	SAU00171	17	0.743	1.941	0.0519	0.7442	220–250
41	SAU00172	2	0.453	0.611	0.3043	0.4218	185–190
42	SAU00176	19	0.834	2.256	0.4490	0.8450	205–232
43	SAU00180	8	0.694	1.332	0.1034	0.6934	260–279
	Mean	9.21	0.606	1.294	0.1757	0.5935	

**All number are the number of alleles detected by each marker among 155 accessions. PIC = polymorphism information content for each marker. I = Shannon’s Information index (Lewontin 1972). Ho is observed heterozygosity, and He is expected heterozygosity that was computed using Levene 1949.**

Pairwise genetic similarity coefficients were calculated among all samples which are provided in Supplementary Table S9. The mean genetic similarity coefficient among these accessions was 0.8642 (range: 0.7828–1.000). The least genetic similarity coefficient was 0.7928 between “Suqian 1-C” and US4 suggesting they are genetically the most distant among these accessions. Meanwhile, the genetic similarity coefficient between ‘Datonghuanghua’ (H0006) and ‘Qiaotouhuanghua’ (H0010) was 1.000 indicating they may actually belong to the same accession.

A UPGMA dendrogram of the 155 accessions based on 43 EST-SSR markers was constructed (Supplementary Figure S2). A cophenetic correlation test (*r* = 0.8687) showed that the clustering results were credible. Among the 155 accessions, 55 from three species (*H. citrina, H. lilioasphodelus*, and *H. minor*) were clustered in one group (branches highlighted in red in Supplementary Figure S2). Fifty of the 55 accession in this group are known to be night lily accessions, which are represented by ‘Datonghuanghua’ (H0006) and ‘Panlonghua’ (H0004). Thus, this group could be called the “Night Lily Group.” Of the rest 100 accessions, 95 bloomed in the morning, and five [‘Guanglinghuanghua’ (H0011), ‘Stella de Oro 2’ (H0077), ‘Stella de Oro 3’ (H0139), ‘Xue Qiu Hong’ (H0092), and ‘Beijing 6’ (H0101)] open flowers at night (see flowering time data in Supplementary Table S1). These 100 accessions included multiple species such as *H. fulva* [var. *sempervirens* (H0050), and var. *kwanso var. reasata* (H0052)], *H. aurantiaca* (H0040), *H. middendorffii* (H0041), *H. thunbergii* (H0042), *H. multiflora* (H0045), *H. altissima* (H0046), and *H. dumortieri* (H0048) (Supplementary Table S1).

**FIGURE 7:**
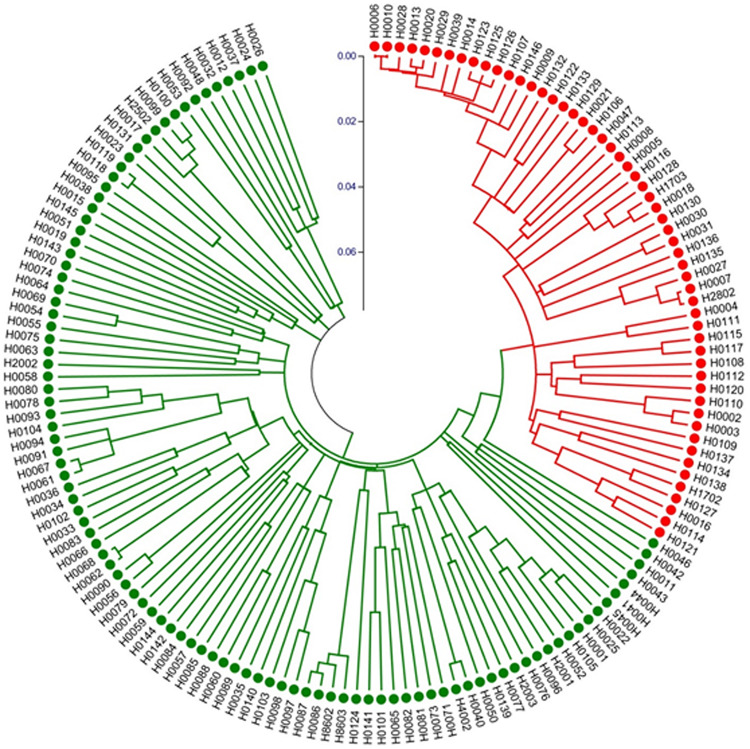
Neighbor-Joining tree of 155 *Hemerocallis* accessions based on 43 EST-SSR constructed using MEGA. Group I (branches in red) includes 55 accessions belonging mostly to the night lily group. Group II (branches in green) consisted of 100 accessions forming the daylily group.

We also constructed a Neighbor-Joining (NJ) tree for the 155 accessions, which is presented in Figure 7. The groupings were largely the same as the UPGMA dendrogram (Supplementary Figure S2) with all night lily accession in one clade. These data suggested that night lily has narrower genetic base than daylily, and the night lily is likely the result of human selection from daylilies for human consumption during crop evolution.

### Population Structure of 155 *Hemeracallis* Accessions

**FIGURE 8:**
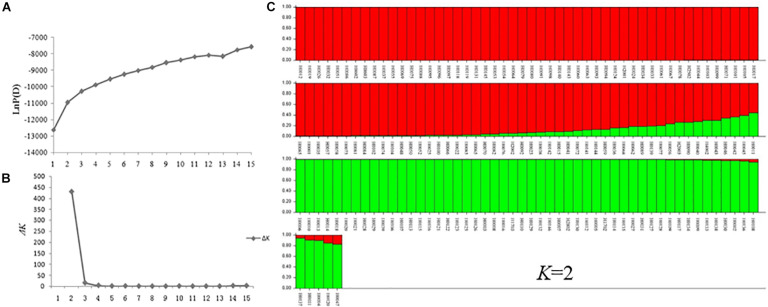
STRUCTURE analysis of 155 *Hemerocallis* accessions. **(A)** The relationship between *K*-value and LnP(D) value. **(B)** The relationship between *K-*value and Δ*K* value. **(C)** Clustering of 155 *Hemerocallis* accessions. The red is Groups II consisting of 102 accessions (daylily group), the green is Group I including 53 accessions (night lily group).

A model-based Bayesian clustering approach was used to analyze 155 accessions by STRUCTURE. The logarithm of the likelihood [Ln P(D)] on average continued to increase with increasing numbers of assumed subpopulations (*K*) from 2 to 20 (Figure 8A). Difference between Ln P(D) values at two successive *K*-values became non-significant after *K* = 5. As the *ad hoc* statistic Δ*K* preferentially detects the uppermost level of structure and gave the highest value at *K* = 2 (Figure 8B; Lia et al., 2009; Emanuelli et al., 2013). Thus, *K* = 2 was considered as the most probable prediction for the number of subpopulations. Indeed, in the first round of structure analysis, the 155 accessions were split into two major clusters. Group I contained 53 accessions with an average Q value of 0.9867 (86.79% of them with Q > 0.98), and Group II contained 102 accessions with an average Q value of 0.9213 (78.43% of them with Q > 0.90) (Figure 8C and Supplementary Table S1).

Group I consisted of 53 accessions, all of which were blooming at night (night lily group). Groups II consisted of 102 accessions, 97 accessions of them were blooming in the morning (daylily group) (Supplementary Table S1). Some accessions were admixtures of the two groups.

### Association Analysis of Blooming Time Among 155 Accessions

Blooming time is a very important horticultural trait for both daylily and night lily varieties. We recorded blooming time of all 155 accessions, and the data were presented in Supplementary Table S1. Among them, 96 bloomed (almost all daylily) in the morning (7–10 a.m.), and the rest (almost night lily) in the later afternoon or evening (4–11:30 p.m.). To confirm the utility of the EST-SSR markers developed in the present study, we conducted association analysis of blooming time using these markers. We first examined linkage disequilibrium (LD) in the *Hemerocallis* population. Of the 903 pairwise combinations generated for 43 the EST-SSR loci, 786 (87.0%) showed LD (Supplementary Table S10 and Supplementary Figure S3). At *P* ≤ 0.001, 74 pairs (8.2%) had mean *r*^2^ of 0.4266 indicating strong LD. At *P* ≤ 0.001, the standardized disequilibrium coefficients (D’) was 0.8435 which was close to 1.0 suggesting low chance of recombination between pairs of loci, which may be caused by the long-term asexual reproduction among the *Hemerocallis* collection.

**FIGURE 9:**
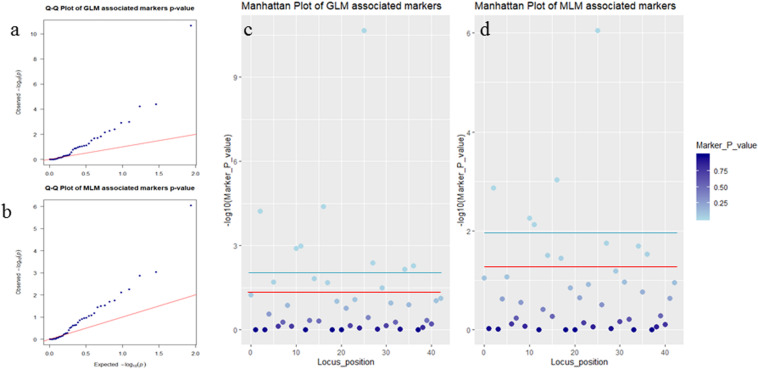
Association analysis of SSR markers with flower timing among 155 *Hemerocallis* accessions. In **(a,b)**, QQ plots showing the *P* value of associated markers deviated from the expected *P-*value in GLM Model **(a)** and MLM Model **(b)**. Blue dots and red lines indicate the *P-*value of the associated markers, and the expected *P-*value, respectively. In **(c,d)**, Manhattan plots showed markers associated with flowering time in GLM Model **(c)** and MLM Model **(d)**. Threshold: the red line is *P* = 0.05 and the blue line is *P* = 0.01.

**FIGURE 10:**
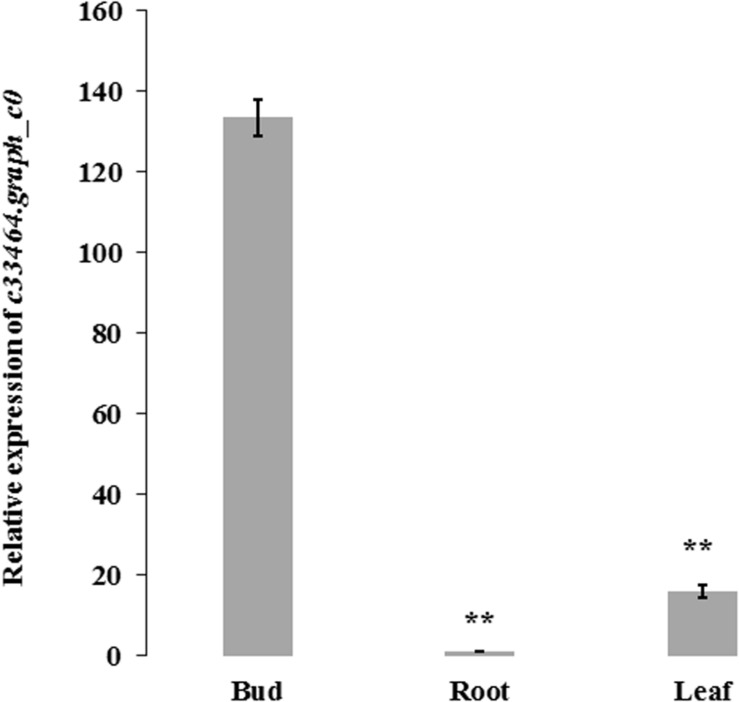
The expression of *c33464.graph_c0* based on quantitative real-time PCR and RNA-Seq in bud, leaf and root of *H. citrina* cv. ‘Datonghuanghua’. The results show the mean ± SD (error bars) and were generated from three biological replicates. ** mean significantly difference, *P* ≤ 0.01. *HcACT* was used as an internal control for RT-qPCR.

Association analysis was performed using both the general linear model (GLM, Q model) and the mixed linear model (MLM, Q+K model) in TASSEL. The results are presented in Table 5, and illustrated in Figure 9. In the GLM model, 12 markers showed significant association with blooming time, 8 of which had extremely high significance (*P* ≤ 0.01; mean *r*^2^ = 0.0459) (Points above the blue line in Figure 9c). Similarly, under the MLM model, 10 markers were significantly associated with blooming time and 5 had extremely significant association (*P* ≤ 0.01; mean *r*^2^ = 0.0560) (Points above the blue line in Figure 9d). The 10 markers with significant associations with the blooming time in both GLM and MLM were SAU00008, SAU00045, SAU00047, SAU00045, SAU00063, SAU00064, SAU00109, SAU00121, SAU00143, and SAU00150. Among them, SAU00109 had the strongest association (*P* << 0.001 in MLM model) that could explain 8.8% phenotypic variance (8.0% in GLM) (Table 5). The predicted functions of the 12 unigenes associated with these SSRs are provided in Table 5. Both SAU00064 and SAU00109 were associated with the same unigenes *c22731.graph_c0*, which seems to encode a eukaryotic translation initiation factor 2D. Another unigene, *c33464.graph_c0*, associated with marker SAU00063 was annotated to encode a LHY-like protein, which is known to be regulated by circadian clock. The expression of *c33464.graph_c0* in flower buds was significantly higher than that of roots and leaves in both RNA-Seq and RT-qPCR experiments (Figure 10). The unigene *c47752.graph_c0* associated with SAU00008 was predicted to encode phosphatase 2C 35/65, and its expression level in roots was significantly different from that in flower buds and leaves.

**TABLE 5:** Twelve SSR markers with significant association with blooming time in 155 *Hemerocallis* accessions*.

Markers with Significant association	R2		Unigenes Associated with markers	Predicted functions of unigenes
	GLM	MLM		
SAU00008	0.05538**	0.06179**	*c47752.graph_c0*	Protein phosphatase 2C 35
SAU00014	0.04321*		*c50474.graph_c0*	29 kDa ribonucleoprotein B
SAU00045	0.03733**	0.04143**	*c17410.graph_c0*	F-box protein PP2-A15
SAU00047	0.04347**	0.04721**	*c18505.graph_c0*	Pre-mRNA-processing protein 40A isoform X1
SAU00055	0.02924*	0.03254*	*c24436.graph_c0*	Uncharacterized protein LOC105044077
SAU00063	0.04051**	0.04161**	*c33464.graph_c0*	Protein LHY-like
SAU00064	0.02803*	0.03182*	*c22731.graph_c0*	Eukaryotic translation initiation factor 2D isoform X1
SAU00109	0.08038**	0.08781**	*c22731.graph_c0*	Eukaryotic translation initiation factor 2D isoform X1
SAU00121	0.0373**	0.04022*	*c52364.graph_c0*	bZIP transcription factor 60
SAU00132	0.0194*		*c20852.graph_c0*	protein-tyrosine-phosphatase MKP1-like
SAU00143	0.02814**	0.03023*	*c19709.graph_c0*	Basic proline-rich protein-like isoform X2
SAU00150	0.04503**	0.04799*	*c28194.graph_c0*	Myosin IB heavy chain-like isoform X1

* P ≤ 0.05; ** P ≤ 0.01.

## Discussion

In this study, 92,107 *Hemerocallis* transcriptomic unigenes were assembled using the Illumina Hi-Seq 2500 platform (Table 1). The N50 length of these unigenes was 908 bp, and the average length was 575 bp. These results were comparable to what obtained in other recent studies in species of the Liliaceae family, such as *Lilium regale* (N50 = 920 bp, average length = 682 bp) (Cui et al., 2018). The number of unigenes and contained SSRs obtained from the present study (92,107 unigenes with 3,430 SSRs) was also close the number identified in other three Liliaceae species (*L. formolong, L. longiflorum*, and *L. longiflorum*) (average unigenes = 72,256) (Biswas et al., 2018). These results suggested that closely related species share similar gene contents and distribution of SSR sequences in their genomes.

Fingerprinting is among the most popular uses of molecular markers. In *Hemerocallis*, hundreds of varieties have been released. Many varieties have the same names but may be morphologically different, or have the same appearances but with different names. Marker-based fingerprinting can help resolve these issues. In the study, the two landraces ‘Malinhuanghua (H0029)’ and ‘Malinhuanghua2 (H0125)’ had the same name (in Chinese), but have different geographic origins. Clustering analysis indicated that they were located in different clades (Figure 7 and Supplementary Figure S2). Therefore, these two accessions do not seem to share any common parent, but may have the same name by chance. The three accessions, ‘Taiguxuancao (H0038),’ ‘Stella de Oro 2 (H0077),’ and ‘Stella de Oro 3 (H0139)’ may have a similar situation. In addition, three accessions, *H. minor* Mill. (H0007) and *H. minor* Mill.2 (H2802) were all introduced from Qingyang, Gansu Province, China, but clustering analysis did not support their close relatedness. These apparent mis-identifications were probably because the original names of these foreign varieties were changed right after their introduction. This is also likely true for many accessions that were introduced from one place to another place inside China. Work in the present study presents a good example to show the power of molecular markers in variety identification and protection.

Both clustering analysis and STRUCTURE analysis place ∼50 accessions into one group (Figure 7 and Supplementary Figure S2) that bloom in the evening, while the rest of ∼100 accession all bloom in the morning (Supplementary Table S1). Marker-based phylogenetic analysis revealed that the night lily accessions were more genetically related, which seems to be consistent with their geographical distribution. Accessions in the daylily group were collected from more geographically diverse locations (Supplementary Table S1). Based on our multiple observations (Ji et al., 2018), accessions in the daylily group are also morphologically more diverse than those in the night lily group. For example, the colors of petals and sepals of daylilies may vary from yellow, yellow-orange, orange, orange-red, red, purple-red to purple. For flower size, the width variation coefficient was high in petals and sepals. Among them, the orange petal width variation coefficient was the highest of 42.15% and the average width was 35.90 mm (13.58–75.56 mm). Similarly, the variation coefficient of the orange sepal width was the highest at 38.74%, and the average width was 23.40 mm (13.20∼48.34 mm). The difference between stalk height and leaf width were highly significant. The inflorescences were rich in morphological variation ranging from mini inflorescence, extremely short branches, to large branched inflorescence, with varying amounts of blossoms. On the other hand, the 55 accessions in the night lily group (branches in red in Figure 7 and Supplementary Figure S2 were collected from nine provinces of China, all of which were diploids and are edible. For these accessions, both petals and sepals were yellow while the width of petals and sepals were very small, with the mean values 17.46 and 13.77 mm, respectively. As compared with accessions in the daylily group, the stalk height (main stem) of night lily group was in general higher (129.08 cm), the leaf was narrower (3.16 cm), and the average number of blossoms was 22.10. Fifty-three accessions were blooming at night, except for ‘Panlonghua’ (H0004) and ‘Panlonghua 2’ (H0111) that bloomed in the morning. The results from both marker analysis (Figure 7 and Supplementary Figure S2) and morphological observations indicate that the night lily group may have narrower genetic base than the daylily group accessions in this collection. This seems reasonable because the daylily group accessions had a more diverse geographic origin (Supplementary Table S1). Also, many of the night lily accessions may share common parents during their breeding and selection. From the crop evolution perspective, night lily was probably selected from the genetically more diverse daylily gene pool for vegetable use. During this process, blooming at night may be a main target of selection which is critical for vegetable use to collect the tender unopened flower bud during day time.

A few accessions were placed in different clades when different programs were used in clustering analysis, for example, ‘Panlonghua’ and ‘Panlonghua 2’ were clustered into night lily group by NTSYS and MEGA, which were in daylily group by STRUCTURE (Supplementary Table S1, Figures 7, 8, and Supplementary Figure S2). ‘Panlonghua’ and ‘Panlonghua 2’ were edible landraces, which were probably the hybrids between the night lily and the daylily. They displayed characteristics of both parents including morning blooming and the narrow perianth traits. ‘Stella de Oro 2’ (H0077), ‘Xue Qiu Hong’ (H0092), ‘Beijing 6’ (H0101), and ‘Little Bumble Bee’ (H0141) were daylily varieties that blossom at night, but they were clustered together with other daylily accessions that bloomed during the day. These observations suggest gene flow between the two cultivated groups during the long-term breeding and selection. *Hermerocallis* varieties are often recognized as either daylily or night lily based on their blooming time. Probably only a few genes with strong phenotypic effects are underlying the blooming time (Masahiro et al., 2006). In this study, we identified 12 SSRs with strong association with blooming time (Table 5). The SSR marker SAU00063 with the strongest association is in the unigene (*c33464.graph_c0*) that was annotated to encode a LHY-like protein (Table 5). The *LHY* (*Late Elongated Hypocotyl*) gene has been shown to regulate flower timing in a number of plants (e.g., Ramos-Sanchez et al., 2019; Zhang et al., 2019). Our work in the present study provides a foundation for gene discovery of flower timing in *Hermerocallis*. However, additional work is needed to validate this result.

## Conclusion

This is the first report on the *Hemerocallis* transcriptome, and a large number of EST-SSR markers have been developed from this transcriptome, which will provide an excellent resource for researchers and breeders focusing on *Hemerocallis*. More importantly, our results showed that this strategy using high-throughput sequencing technology is feasible and extremely convenient and efficient in the genus *Hemerocallis*. The genes associated with circadian rhythm provide germplasm resources and genes basis for further study on flowering rhythm of this genus.

## Data Availability Statement

The datasets presented in this study can be found in NCBI under accession numbers SRR11610941, SRR11610942, and SRR11610943 (Submission ID: SUB7327030; BioProject ID: PRJNA628147).

## Author Contributions

SL designed and conducted the whole research. FJ collected the phenotype data and conducted the GWAS analysis. FH collected some accessions and developed the EST-SSR. HC analyzed the transcriptome data. QS ran the PAGE gel. GX and XK kept the Hemerocallis germplasm. YW advised the project and revised the manuscript.

## Conflict of Interest

The authors declare that the research was conducted in the absence of any commercial or financial relationships that could be construed as a potential conflict of interest.

## Footnotes

1 http://www.daylilies.org/

2 http://trinityrnaseq.sourceforge.net/

3 http://pgrc.ipk-gatersleben.de/misa/misa.html

4 http://bioinfo.ut.ee/primer3-0.4.0/

5 https://github.com/egonozer/in_silico_pcr/
