# Supplementary material for: Characterization of Hemerocallis citrina Transcriptome and Development of EST-SSR Markers for Evaluation of Genetic Diversity and Population Structure of Hemerocallis Collection
Source: Front Plant Sci. 2020 Jun 11;11:686. doi: 10.3389/fpls.2020.00686 (PMC7300269; doi:10.3389/fpls.2020.00686)
Supplement: Supplementary file 2 [file Presentation_1.PPTX]

## Slide 1
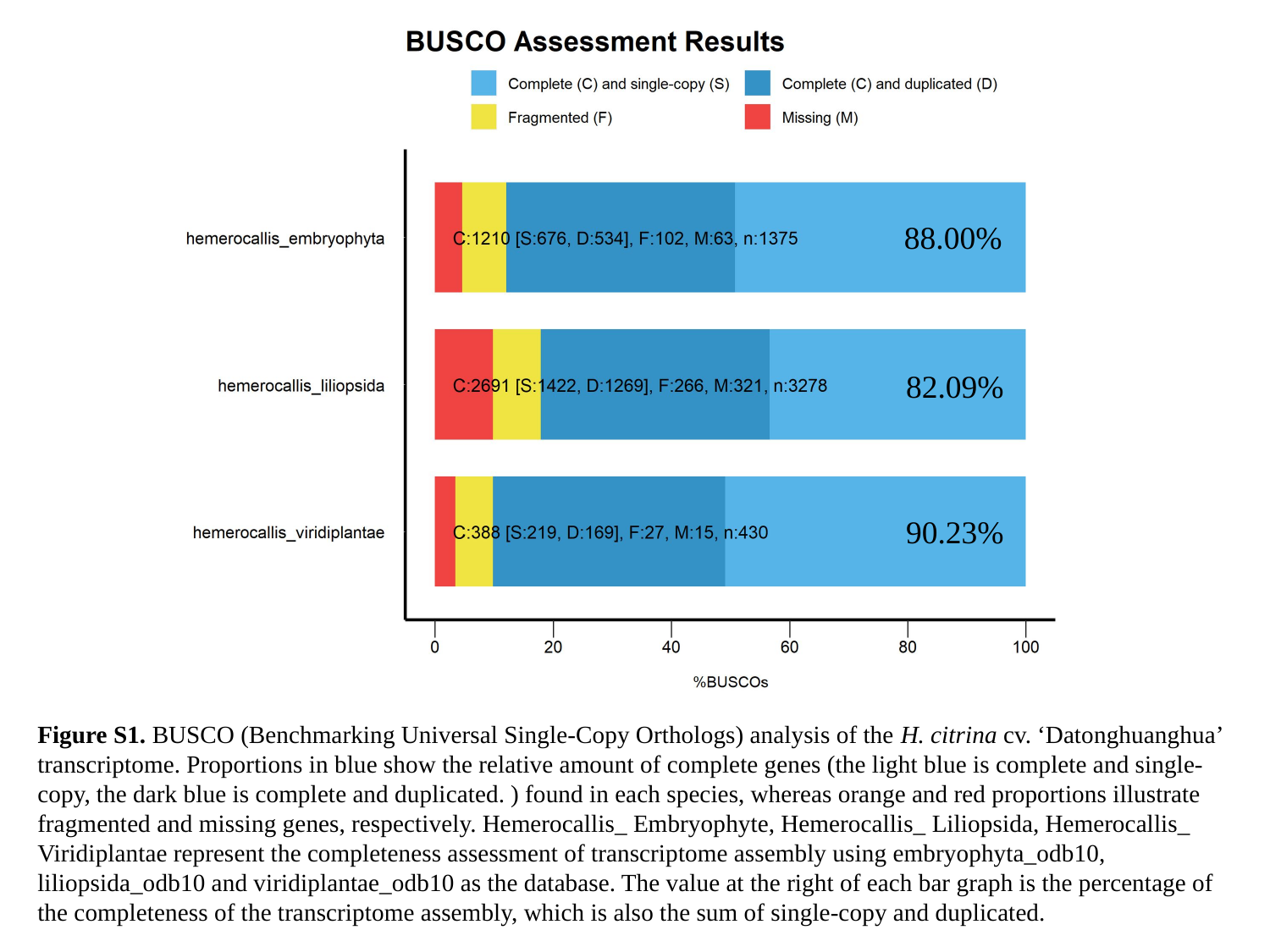

88.00%
82.09%
90.23%
# Figure S1. BUSCO (Benchmarking Universal Single-Copy Orthologs) analysis of the H. citrina cv. ‘Datonghuanghua’ transcriptome. Proportions in blue show the relative amount of complete genes (the light blue is complete and single-copy, the dark blue is complete and duplicated. ) found in each species, whereas orange and red proportions illustrate fragmented and missing genes, respectively. Hemerocallis_ Embryophyte, Hemerocallis_ Liliopsida, Hemerocallis_ Viridiplantae represent the completeness assessment of transcriptome assembly using embryophyta_odb10, liliopsida_odb10 and viridiplantae_odb10 as the database. The value at the right of each bar graph is the percentage of the completeness of the transcriptome assembly, which is also the sum of single-copy and duplicated.

## Slide 2
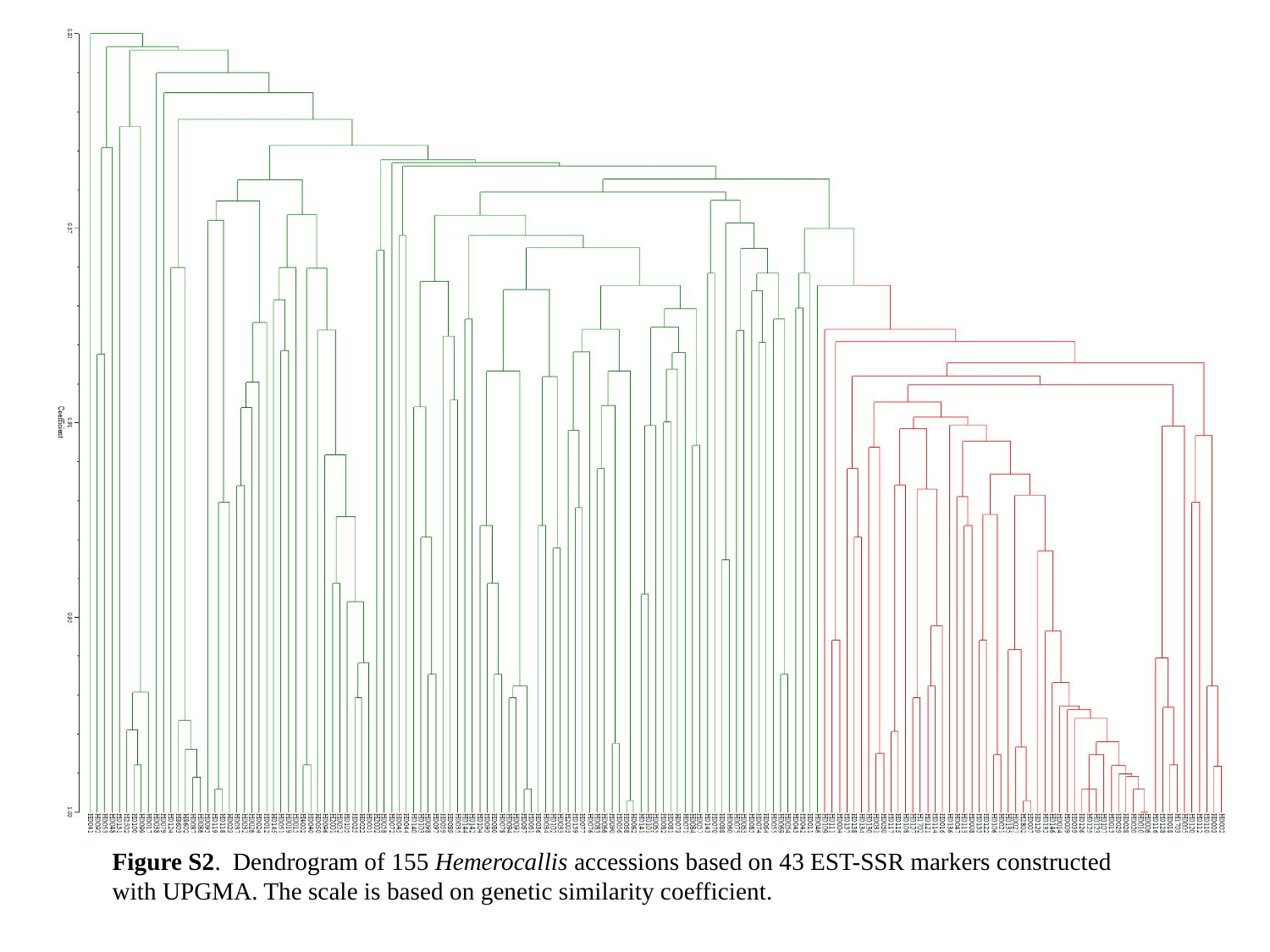

Figure S2. Dendrogram of 155 Hemerocallis accessions based on 43 EST-SSR markers constructed with UPGMA. The scale is based on genetic similarity coefficient.

## Slide 3
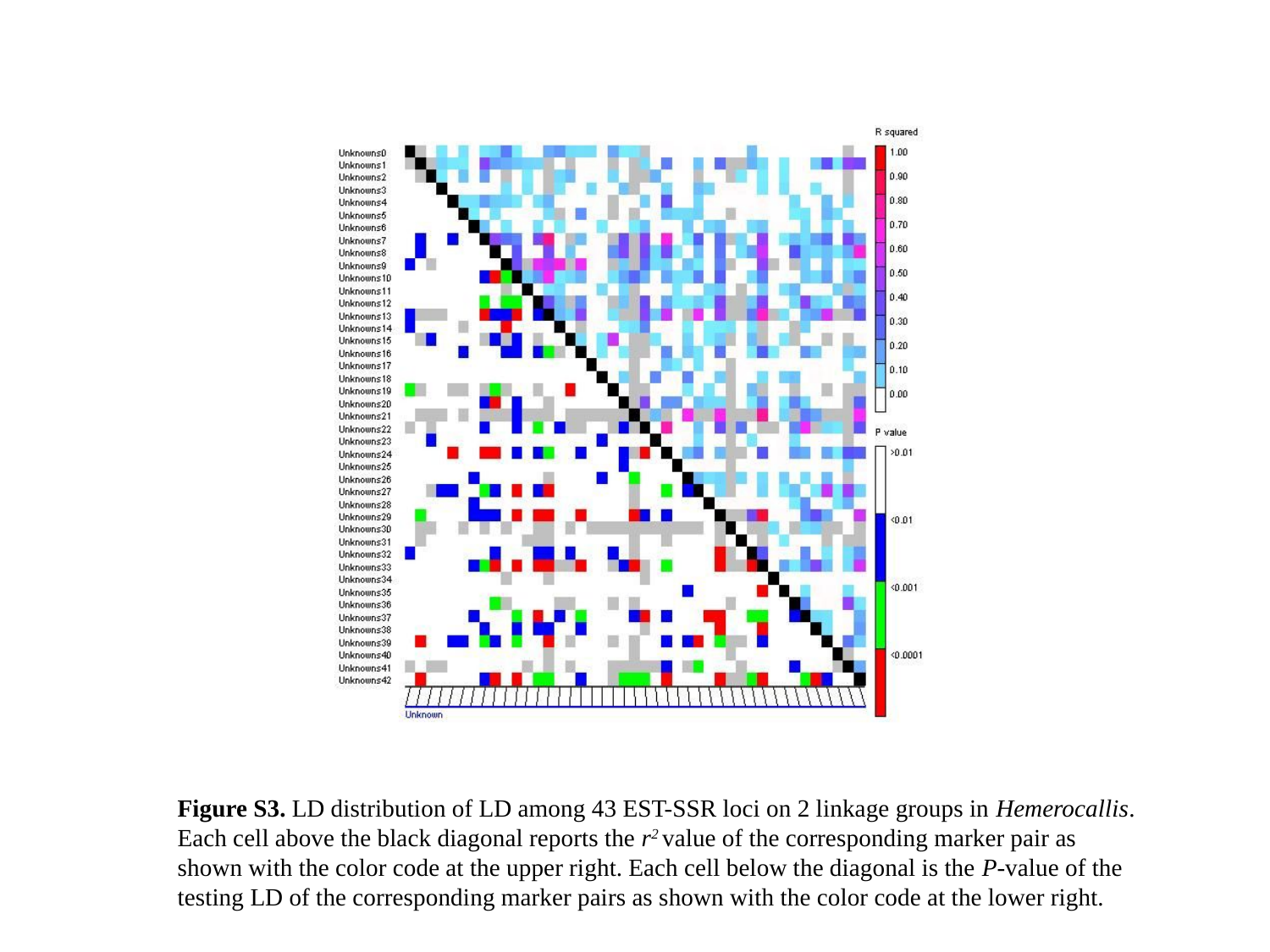

Figure S3. LD distribution of LD among 43 EST-SSR loci on 2 linkage groups in Hemerocallis. Each cell above the black diagonal reports the r2 value of the corresponding marker pair as shown with the color code at the upper right. Each cell below the diagonal is the P-value of the testing LD of the corresponding marker pairs as shown with the color code at the lower right.
